# (4-Fluoro­phen­yl)(4-hy­droxy-3-methyl­phen­yl)methanone

**DOI:** 10.1107/S1600536813033783

**Published:** 2013-12-21

**Authors:** C. S. Dileep, V. Lakshmi Ranganatha, N. K. Lokanath, S. A. Khanum, M. A. Sridhar

**Affiliations:** a Department of Studies in Physics, Manasagangotri, University of Mysore, Mysore 570 006, India; b Department of Chemistry, Yuvaraja’s College, University of Mysore, Mysore 570 005, India

## Abstract

In the title compound, C_14_H_11_FO_2_, the two benzene rings are not coplanar, with a dihedral angle of 57.45 (12)° between their planes. In the crystal, mol­ecules are linked by an O—H⋯O hydrogen bond, forming a 2_1_ helical chain along the *b* axis.

## Related literature   

For the biological activities of benzo­phenone derivatives, see: Khanum *et al.* (2004 ▶); Naveen *et al.* (2006 ▶); Selvi *et al.* (2003 ▶). For related structures, see: Mahendra *et al.* (2005 ▶); Dileep, Lakshmi Ranganatha *et al.* (2013 ▶); Dileep, Prashanth *et al.* (2013 ▶). For bond-length data, see: Allen *et al.* (1987 ▶).
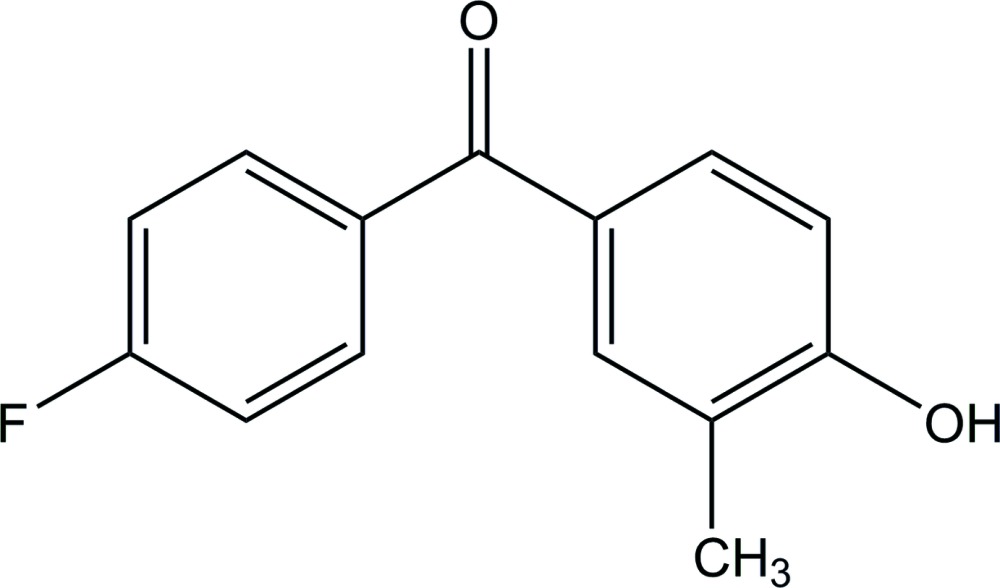


## Experimental   

### 

#### Crystal data  

C_14_H_11_FO_2_
*M* *_r_* = 230.23
Monoclinic, 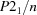

*a* = 5.9265 (10) Å
*b* = 13.112 (2) Å
*c* = 14.556 (2) Å
β = 96.875 (7)°
*V* = 1123.0 (3) Å^3^
*Z* = 4
Cu *K*α radiation
μ = 0.85 mm^−1^
*T* = 296 K
0.27 × 0.25 × 0.23 mm

#### Data collection  

Bruker X8 Proteum diffractometer
Absorption correction: multi-scan (*SADABS*; Bruker, 2009 ▶) *T*_min_ = 0.804, *T*_max_ = 0.829
7047 measured reflections
1769 independent reflections
1317 reflections with *I* > 2σ(*I*)
*R*_int_ = 0.065

#### Refinement  

*R*[*F* ^2^ > 2σ(*F* ^2^)] = 0.068
*wR*(*F* ^2^) = 0.273
*S* = 1.16
1769 reflections
155 parameters
H-atom parameters constrained
Δρ_max_ = 0.27 e Å^−3^
Δρ_min_ = −0.36 e Å^−3^

### 

Data collection: *APEX2* (Bruker, 2009 ▶); cell refinement: *SAINT* (Bruker, 2009 ▶); data reduction: *SAINT*; program(s) used to solve structure: *SHELXS97* (Sheldrick, 2008 ▶); program(s) used to refine structure: *SHELXL97* (Sheldrick, 2008 ▶); molecular graphics: *Mercury* (Macrae *et al.*, 2006 ▶); software used to prepare material for publication: *SHELXL97*.

## Supplementary Material

Crystal structure: contains datablock(s) global, I. DOI: 10.1107/S1600536813033783/is5325sup1.cif

Structure factors: contains datablock(s) I. DOI: 10.1107/S1600536813033783/is5325Isup2.hkl

Supporting information file. DOI: 10.1107/S1600536813033783/is5325Isup3.cml

Additional supporting information:  crystallographic information; 3D view; checkCIF report

## Figures and Tables

**Table 1:** Hydrogen-bond geometry (Å, °)

*D*—H⋯*A*	*D*—H	H⋯*A*	*D*⋯*A*	*D*—H⋯*A*
O14—H14⋯O9^i^	0.82	1.91	2.688 (3)	158

Symmetry code: (i) 
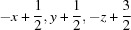
.

**Fig. 1.**
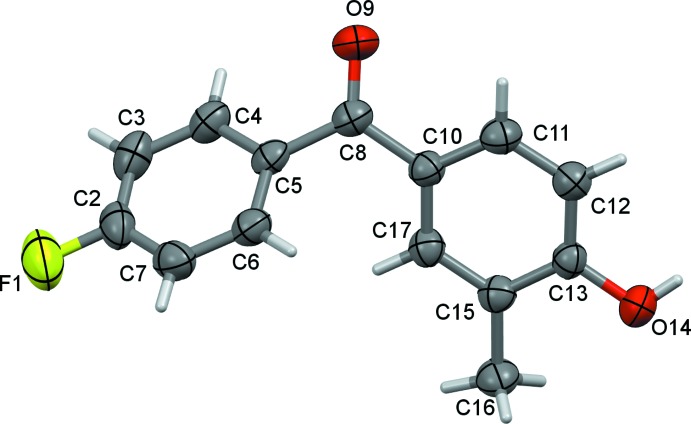
An ORTEP view of the title compound with the atom-labeling scheme. The thermal ellipsoids are drawn at the 50% probability level.

**Fig. 2.**
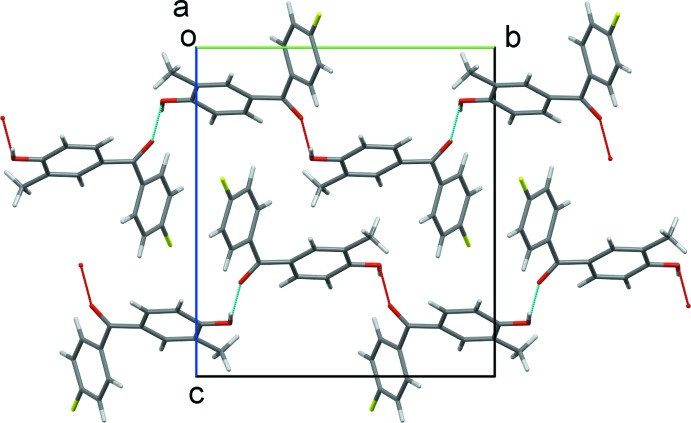
A molecular packing view of the title compound down the a axis, showing hydrogen bonds between the molecules.

## supplementary crystallographic information

### 1. Comment

The great interest in the benzophenone substances is fundamentally due to their diverse biological and chemical properties. Benzophenone and related compounds have a wide variety of biological activities such as anti-fungal and anti-inflammatory activities (Khanum *et al.*, 2004; Selvi *et al.*, 2003). The presence of various substituents in the benzophenone nucleus is essential in determining the quantitative structure-activity relationships of these systems. The competence of benzophenones as chemotherapeutic agents, especially as inhibitors of HIV-1 reverse transcriptase RT, cancer and inflammation, is well established. Their chemistry has been studied extensively. In addition, methyl-substituted benzophenones exhibit chemotherapeutical activity against fungi. Some studies were carried out to show that methyl-substituted benzophenones exhibit anti-fungal properties (Naveen *et al.*, 2006). In view of its extensive background, the title compound was prepared and characterized by single-crystal X-ray diffraction.

In the molecular structure of the title compound (Fig. 1), bond lengths and angles do not show large deviations and are comparable with those reported for a similar structure (Mahendra *et al.*, 2005; Dileep, Lakshmi Ranganatha *et al.*, 2013; Dileep, Prashanth *et al.*, 2013). The mean plane angle between the two phenyl rings (C2–C7) and (C10–C13/C15/C17) is 57.45 (12)°. The bond length between C2 and F1 is 1.357 (4) Å and is normal with the standard value (Allen *et al.*, 1987). The conformation of the attachment of the two phenyl rings to the central carbonyl group can also be characterized by torsion angles (O9—C8—C5—C6) and (O9—C8—C10—C17) of -141.1 (3) and -152.8 (3)°, respectively. The crystal structure is stabilized by intermolecular O—H···O hydrogen bonds. The molecular packing when viewed down the *a* axis is shown in Fig. 2.

### 2. Experimental

The title compound was synthesized by a mixture of anhydrous aluminium chloride (0.03 mol) and 2-methyl-phenyl-4-fluorobenzoate (0.02 mol) in dry nitrobenzene (40 ml) was protected from moisture by calcium chloride guard tube and refluxed at 80–900 °C with stirring for 45 min. At the end of this period the solution was cooled and decomposed by acidulated ice-cold water. Nitrobenzene was removed by steam distillation. The residual solid was crushed into powder, dissolved in ether and extracted with 10 percent sodium hydroxide. The basic aqueous solution was neutralized with 10 percent hydrochloric acid. The filtered solid was washed with distilled water and recrystallized from ethanol to afford pale yellow needles of (4-fluorophenyl)(4-hydroxy-3-methylphenyl)methanone.

### 3. Refinement

All H-atoms were located from difference maps and were positioned geometrically and refined using a riding model with C—H = 0.93–0.96 Å and O—H = 0.82 Å, and with *U*_iso_(H) = 1.2*U*_eq_(C_aromatic_) or 1.5*U*_eq_(O, C_methyl_). The data collection did not yield reflections with measurable intensity range as the crystal was diffracting a bit poorly. Hence, the range is slightly less (64.45° rather than the required 65°).

### Crystal data

**Table:**

C_14_H_11_FO_2_	*F*(000) = 480
*M**_r_* = 230.23	*D*_x_ = 1.362 Mg m^−^^3^
Monoclinic, *P*2_1_/*n*	Cu *K*α radiation, λ = 1.54178 Å
Hall symbol: -P 2yn	Cell parameters from 1769 reflections
*a* = 5.9265 (10) Å	θ = 4.6–64.5°
*b* = 13.112 (2) Å	µ = 0.85 mm^−^^1^
*c* = 14.556 (2) Å	*T* = 296 K
β = 96.875 (7)°	Block, colorless
*V* = 1123.0 (3) Å^3^	0.27 × 0.25 × 0.23 mm
*Z* = 4	

### Data collection

**Table:**

Bruker X8 Proteum diffractometer	1769 independent reflections
Radiation source: Bruker MicroStar microfocus rotating anode	1317 reflections with *I* > 2σ(*I*)
Helios multilayer optics monochromator	*R*_int_ = 0.065
Detector resolution: 10.7 pixels mm^-1^	θ_max_ = 64.5°, θ_min_ = 4.6°
φ and ω scans	*h* = −3→6
Absorption correction: multi-scan (*SADABS*; Bruker, 2009)	*k* = −15→15
*T*_min_ = 0.804, *T*_max_ = 0.829	*l* = −16→16
7047 measured reflections	

### Refinement

**Table:**

Refinement on *F*^2^	Primary atom site location: structure-invariant direct methods
Least-squares matrix: full	Secondary atom site location: difference Fourier map
*R*[*F*^2^ > 2σ(*F*^2^)] = 0.068	Hydrogen site location: inferred from neighbouring sites
*wR*(*F*^2^) = 0.273	H-atom parameters constrained
*S* = 1.16	*w* = 1/[σ^2^(*F*_o_^2^) + (0.197*P*)^2^] where *P* = (*F*_o_^2^ + 2*F*_c_^2^)/3
1769 reflections	(Δ/σ)_max_ = 0.007
155 parameters	Δρ_max_ = 0.27 e Å^−^^3^
0 restraints	Δρ_min_ = −0.36 e Å^−^^3^

### Special details

**Table:**

Geometry. Bond distances, angles *etc*. have been calculated using the rounded fractional coordinates. All su's are estimated from the variances of the (full) variance-covariance matrix. The cell e.s.d.'s are taken into account in the estimation of distances, angles and torsion angles
Refinement. Refinement on *F*^2^ for ALL reflections except those flagged by the user for potential systematic errors. Weighted *R*-factors *wR* and all goodnesses of fit *S* are based on *F*^2^, conventional *R*-factors *R* are based on *F*, with *F* set to zero for negative *F*^2^. The observed criterion of *F*^2^ > σ(*F*^2^) is used only for calculating -*R*-factor-obs *etc*. and is not relevant to the choice of reflections for refinement. *R*-factors based on *F*^2^ are statistically about twice as large as those based on *F*, and *R*-factors based on ALL data will be even larger.

### Fractional atomic coordinates and isotropic or equivalent isotropic displacement parameters (Å^2^)

**Table:**

	*x*	*y*	*z*	*U*_iso_*/*U*_eq_	
F1	−0.8701 (4)	0.08743 (17)	0.39982 (15)	0.0892 (9)	
O9	−0.0623 (4)	0.14888 (15)	0.71425 (13)	0.0602 (8)	
O14	0.1969 (4)	0.61296 (12)	0.66191 (12)	0.0526 (8)	
C2	−0.6901 (6)	0.1166 (2)	0.4605 (2)	0.0549 (10)	
C3	−0.6834 (6)	0.0875 (2)	0.5518 (2)	0.0565 (10)	
C4	−0.4989 (5)	0.11772 (18)	0.61269 (17)	0.0480 (10)	
C5	−0.3285 (5)	0.17959 (17)	0.58441 (15)	0.0402 (8)	
C6	−0.3409 (5)	0.20673 (18)	0.49111 (16)	0.0464 (9)	
C7	−0.5214 (6)	0.1741 (2)	0.42877 (17)	0.0544 (9)	
C8	−0.1343 (5)	0.21080 (19)	0.65391 (15)	0.0420 (8)	
C10	−0.0411 (5)	0.31539 (18)	0.65178 (16)	0.0386 (8)	
C11	0.1779 (5)	0.3367 (2)	0.69635 (17)	0.0443 (9)	
C12	0.2609 (5)	0.43554 (19)	0.69977 (16)	0.0440 (9)	
C13	0.1231 (5)	0.51463 (17)	0.65931 (14)	0.0385 (8)	
C15	−0.0954 (5)	0.49616 (18)	0.61519 (15)	0.0395 (8)	
C16	−0.2400 (5)	0.5833 (2)	0.57530 (19)	0.0502 (10)	
C17	−0.1731 (5)	0.39631 (17)	0.61220 (15)	0.0397 (8)	
H3	−0.79970	0.04880	0.57170	0.0680*	
H4	−0.48790	0.09630	0.67400	0.0580*	
H6	−0.22750	0.24690	0.47080	0.0560*	
H7	−0.52850	0.19070	0.36640	0.0650*	
H11	0.26740	0.28410	0.72370	0.0530*	
H12	0.40630	0.44940	0.72860	0.0530*	
H14	0.32560	0.61580	0.68970	0.0790*	
H16A	−0.18110	0.60860	0.52110	0.0750*	
H16B	−0.23820	0.63690	0.62030	0.0750*	
H16C	−0.39320	0.56010	0.55900	0.0750*	
H17	−0.31810	0.38260	0.58290	0.0480*	

### Atomic displacement parameters (Å^2^)

**Table:**

	*U*^11^	*U*^22^	*U*^33^	*U*^12^	*U*^13^	*U*^23^
F1	0.0791 (16)	0.0866 (15)	0.0952 (15)	−0.0148 (13)	−0.0165 (15)	−0.0221 (11)
O9	0.0725 (17)	0.0477 (12)	0.0571 (11)	−0.0012 (11)	−0.0052 (12)	0.0163 (8)
O14	0.0604 (15)	0.0388 (12)	0.0564 (12)	−0.0082 (9)	−0.0018 (11)	−0.0009 (7)
C2	0.0504 (19)	0.0456 (16)	0.0657 (16)	−0.0019 (15)	−0.0059 (16)	−0.0159 (13)
C3	0.053 (2)	0.0429 (15)	0.0758 (18)	−0.0103 (16)	0.0174 (17)	−0.0100 (13)
C4	0.056 (2)	0.0390 (15)	0.0514 (14)	−0.0040 (13)	0.0167 (15)	0.0008 (10)
C5	0.0481 (17)	0.0279 (12)	0.0455 (12)	0.0003 (12)	0.0088 (13)	−0.0005 (9)
C6	0.055 (2)	0.0371 (14)	0.0478 (13)	−0.0018 (13)	0.0087 (15)	0.0038 (9)
C7	0.072 (2)	0.0454 (15)	0.0437 (13)	0.0038 (16)	−0.0011 (16)	−0.0018 (10)
C8	0.0462 (17)	0.0369 (13)	0.0437 (12)	0.0020 (12)	0.0087 (13)	0.0028 (9)
C10	0.0408 (17)	0.0354 (13)	0.0393 (11)	0.0018 (11)	0.0038 (12)	0.0004 (9)
C11	0.0446 (18)	0.0412 (14)	0.0473 (13)	0.0052 (13)	0.0058 (13)	0.0038 (9)
C12	0.0443 (17)	0.0438 (15)	0.0435 (13)	0.0000 (13)	0.0034 (14)	0.0001 (10)
C13	0.0428 (16)	0.0360 (13)	0.0367 (11)	−0.0039 (12)	0.0052 (12)	−0.0038 (8)
C15	0.0451 (18)	0.0356 (13)	0.0380 (12)	0.0042 (12)	0.0064 (12)	−0.0029 (9)
C16	0.052 (2)	0.0404 (15)	0.0571 (15)	0.0081 (14)	0.0018 (16)	0.0004 (10)
C17	0.0392 (17)	0.0391 (13)	0.0408 (12)	0.0006 (12)	0.0052 (12)	−0.0041 (9)

### Geometric parameters (Å, º)

**Table:**

F1—C2	1.356 (4)	C12—C13	1.405 (4)
O9—C8	1.234 (3)	C13—C15	1.396 (4)
O14—C13	1.361 (3)	C15—C16	1.503 (4)
O14—H14	0.8200	C15—C17	1.387 (3)
C2—C7	1.376 (5)	C3—H3	0.9300
C2—C3	1.379 (4)	C4—H4	0.9300
C3—C4	1.381 (4)	C6—H6	0.9300
C4—C5	1.395 (4)	C7—H7	0.9300
C5—C6	1.397 (3)	C11—H11	0.9300
C5—C8	1.496 (4)	C12—H12	0.9300
C6—C7	1.385 (4)	C16—H16A	0.9600
C8—C10	1.480 (4)	C16—H16B	0.9600
C10—C17	1.401 (4)	C16—H16C	0.9600
C10—C11	1.408 (4)	C17—H17	0.9300
C11—C12	1.385 (4)		
			
F1···H3^i^	2.7200	C17···H6	2.8300
F1···H12^ii^	2.7200	C17···H16A^vii^	3.0200
O9···O14^iii^	2.688 (3)	H3···F1^i^	2.7200
O14···O9^iv^	2.688 (3)	H4···O9	2.6100
O9···H4	2.6100	H4···O14^v^	2.8300
O9···H12^iii^	2.8600	H4···C13^v^	2.8500
O9···H14^iii^	1.9100	H6···C10	2.8800
O9···H16B^v^	2.8100	H6···C17	2.8300
O9···H11	2.6300	H6···H17	2.5200
O14···H16A	2.8500	H6···O14^vii^	2.6900
O14···H16B	2.6000	H7···C11^xiii^	2.9100
O14···H4^vi^	2.8300	H11···O9	2.6300
O14···H11^iv^	2.7900	H11···O14^iii^	2.7900
O14···H6^vii^	2.6900	H12···H14	2.2900
C2···C3^viii^	3.491 (4)	H12···O9^iv^	2.8600
C2···C4^viii^	3.481 (4)	H12···F1^xiv^	2.7200
C3···C8^ix^	3.594 (4)	H14···H12	2.2900
C3···C2^viii^	3.491 (4)	H14···O9^iv^	1.9100
C4···C2^viii^	3.481 (4)	H14···C8^iv^	3.0100
C6···C17	3.139 (3)	H16A···O14	2.8500
C7···C16^x^	3.479 (4)	H16A···C15^vii^	3.0500
C8···C3^xi^	3.594 (4)	H16A···C17^vii^	3.0200
C16···C7^x^	3.479 (4)	H16B···O14	2.6000
C17···C6	3.139 (3)	H16B···O9^vi^	2.8100
C5···H17	2.6600	H16B···C7^x^	2.9000
C6···H17	2.6600	H16C···H17	2.3900
C7···H16B^x^	2.9000	H16C···H16C^x^	2.5500
C8···H14^iii^	3.0100	H17···C5	2.6600
C10···H6	2.8800	H17···C6	2.6600
C11···H7^xii^	2.9100	H17···H6	2.5200
C13···H4^vi^	2.8500	H17···H16C	2.3900
C15···H16A^vii^	3.0500		
			
C13—O14—H14	109.00	C16—C15—C17	122.3 (3)
F1—C2—C7	118.9 (3)	C13—C15—C17	117.7 (2)
C3—C2—C7	122.5 (3)	C10—C17—C15	122.4 (3)
F1—C2—C3	118.7 (3)	C2—C3—H3	121.00
C2—C3—C4	117.9 (3)	C4—C3—H3	121.00
C3—C4—C5	121.5 (2)	C3—C4—H4	119.00
C4—C5—C6	118.6 (2)	C5—C4—H4	119.00
C4—C5—C8	119.0 (2)	C5—C6—H6	120.00
C6—C5—C8	122.3 (2)	C7—C6—H6	120.00
C5—C6—C7	120.3 (3)	C2—C7—H7	120.00
C2—C7—C6	119.0 (2)	C6—C7—H7	121.00
O9—C8—C10	121.8 (2)	C10—C11—H11	120.00
C5—C8—C10	119.9 (2)	C12—C11—H11	120.00
O9—C8—C5	118.3 (2)	C11—C12—H12	120.00
C8—C10—C11	120.0 (2)	C13—C12—H12	120.00
C8—C10—C17	121.3 (3)	C15—C16—H16A	110.00
C11—C10—C17	118.6 (2)	C15—C16—H16B	109.00
C10—C11—C12	120.4 (2)	C15—C16—H16C	109.00
C11—C12—C13	119.3 (3)	H16A—C16—H16B	109.00
O14—C13—C15	117.0 (2)	H16A—C16—H16C	109.00
C12—C13—C15	121.7 (2)	H16B—C16—H16C	109.00
O14—C13—C12	121.3 (2)	C10—C17—H17	119.00
C13—C15—C16	120.0 (2)	C15—C17—H17	119.00
			
F1—C2—C3—C4	179.9 (2)	C5—C8—C10—C11	−160.8 (2)
C7—C2—C3—C4	−0.1 (4)	C5—C8—C10—C17	24.2 (4)
F1—C2—C7—C6	178.0 (3)	C8—C10—C11—C12	−175.9 (2)
C3—C2—C7—C6	−2.0 (4)	C17—C10—C11—C12	−0.7 (4)
C2—C3—C4—C5	2.8 (4)	C8—C10—C17—C15	175.3 (2)
C3—C4—C5—C6	−3.3 (4)	C11—C10—C17—C15	0.2 (4)
C3—C4—C5—C8	179.3 (2)	C10—C11—C12—C13	0.8 (4)
C4—C5—C6—C7	1.2 (4)	C11—C12—C13—O14	179.3 (2)
C8—C5—C6—C7	178.5 (2)	C11—C12—C13—C15	−0.4 (4)
C4—C5—C8—O9	36.2 (4)	O14—C13—C15—C16	−1.5 (3)
C4—C5—C8—C10	−140.9 (3)	O14—C13—C15—C17	−179.9 (2)
C6—C5—C8—O9	−141.1 (3)	C12—C13—C15—C16	178.2 (2)
C6—C5—C8—C10	41.8 (4)	C12—C13—C15—C17	−0.2 (3)
C5—C6—C7—C2	1.4 (4)	C13—C15—C17—C10	0.3 (4)
O9—C8—C10—C11	22.2 (4)	C16—C15—C17—C10	−178.0 (2)
O9—C8—C10—C17	−152.8 (3)		

Symmetry codes: (i) −*x*−2, −*y*, −*z*+1; (ii) *x*−3/2, −*y*+1/2, *z*−1/2; (iii) −*x*+1/2, *y*−1/2, −*z*+3/2; (iv) −*x*+1/2, *y*+1/2, −*z*+3/2; (v) −*x*−1/2, *y*−1/2, −*z*+3/2; (vi) −*x*−1/2, *y*+1/2, −*z*+3/2; (vii) −*x*, −*y*+1, −*z*+1; (viii) −*x*−1, −*y*, −*z*+1; (ix) *x*−1, *y*, *z*; (x) −*x*−1, −*y*+1, −*z*+1; (xi) *x*+1, *y*, *z*; (xii) *x*+1/2, −*y*+1/2, *z*+1/2; (xiii) *x*−1/2, −*y*+1/2, *z*−1/2; (xiv) *x*+3/2, −*y*+1/2, *z*+1/2.

### Hydrogen-bond geometry (Å, º)

**Table:**

*D*—H···*A*	*D*—H	H···*A*	*D*···*A*	*D*—H···*A*
O14—H14···O9^iv^	0.82	1.91	2.688 (3)	158

Symmetry code: (iv) −*x*+1/2, *y*+1/2, −*z*+3/2.

## References

[bb1] Allen, F. H., Kennard, O., Watson, D. G., Brammer, L., Orpen, A. G. & Taylor, R. (1987). *J. Chem. Soc. Perkin Trans. 2*, pp. S1–19.

[bb2] Bruker (2009). *APEX2*, *SAINT* and *SADABS* Bruker AXS Inc., Madison, Wisconsin, USA.

[bb3] Dileep, C. S., Lakshmi Ranganatha, V., Lokanath, N. K., Shaukath, A. K. & Sridhar, M. A. (2013). *Acta Cryst.* E**69**, o1550. 10.1107/S160053681302521X PMC3790415 24098234

[bb4] Dileep, C. S., Prashanth, T., Jeyaseelan, S., Khanum, S. A. & Sridhar, M. A. (2013). *Acta Cryst.* E**69**, o1676. 10.1107/S1600536813028444 PMC3884333 24454109

[bb5] Khanum, S. A., Venu, T. D., Shashikanth, S. & Firdouse, A. (2004). *Bioorg. Med. Chem. Lett.* **12**, 2093–2095.

[bb6] Macrae, C. F., Edgington, P. R., McCabe, P., Pidcock, E., Shields, G. P., Taylor, R., Towler, M. & van de Streek, J. (2006). *J. Appl. Cryst.* **39**, 453–457.

[bb7] Mahendra, M., Khanum, S. A., Singh, A. K., Shashikanth, S., Doreswamy, B. H., Sridhar, M. A. & Shashidhara Prasad, J. (2005). *Acta Cryst.* E**61**, o2990–o2991.

[bb8] Naveen, S., Khanum, S. A., Devaiah, V. T., Shashikanth, S., Anandalwar, S. M. & Prasad, S. (2006). *Anal. Sci.* **22**, 183–184.

[bb9] Selvi, A. T., Joseph, G. S. & Jayaprakasha, G. K. (2003). *Food Microbiol.* **20**, 455–460.

[bb10] Sheldrick, G. M. (2008). *Acta Cryst.* A**64**, 112–122. 10.1107/S0108767307043930 18156677

